# Gut Microbiome Developmental Patterns in Early Life of Preterm Infants: Impacts of Feeding and Gender

**DOI:** 10.1371/journal.pone.0152751

**Published:** 2016-04-25

**Authors:** Xiaomei Cong, Wanli Xu, Susan Janton, Wendy A. Henderson, Adam Matson, Jacqueline M. McGrath, Kendra Maas, Joerg Graf

**Affiliations:** 1 School of Nursing, University of Connecticut, Storrs, Connecticut, United States of America; 2 Institute for Systems Genomics, University of Connecticut, Farmington, Connecticut, United States of America; 3 Department of Molecular and Cell Biology, University of Connecticut, Storrs, Connecticut, United States of America; 4 Digestive Disorders Unit, Biobehavioral Branch, National Institute of Nursing Research, National Institutes of Health, Bethesda, Maryland, United States of America; 5 Connecticut Children’s Medical Center, Hartford, Connecticut, United States of America; 6 Microbial Analysis, Resources, and Services, University of Connecticut, Storrs, Connecticut, United States of America; University of Palermo, ITALY

## Abstract

Gut microbiota plays a key role in multiple aspects of human health and disease, particularly in early life. Distortions of the gut microbiota have been found to correlate with fatal diseases in preterm infants, however, developmental patterns of gut microbiome and factors affecting the colonization progress in preterm infants remain unclear. The purpose of this prospective longitudinal study was to explore day-to-day gut microbiome patterns in preterm infants during their first 30 days of life in the neonatal intensive care unit (NICU) and investigate potential factors related to the development of the infant gut microbiome. A total of 378 stool samples were collected daily from 29 stable/healthy preterm infants. DNA extracted from stool was used to sequence the V4 region of the 16S rRNA gene region for community analysis. Operational taxonomic units (OTUs) and α-diversity of the community were determined using QIIME software. *Proteobacteria* was the most abundant phylum, accounting for 54.3% of the total reads. Result showed shift patterns of increasing *Clostridium* and *Bacteroides*, and decreasing *Staphylococcus* and *Haemophilus* over time during early life. Alpha-diversity significantly increased daily in preterm infants after birth and linear mixed-effects models showed that postnatal days, feeding types and gender were associated with the α-diversity, p< 0.05–0.01. Male infants were found to begin with a low α-diversity, whereas females tended to have a higher diversity shortly after birth. Female infants were more likely to have higher abundance of *Clostridiates*, and lower abundance of *Enterobacteriales* than males during early life. Infants fed mother’s own breastmilk (MBM) had a higher diversity of gut microbiome and significantly higher abundance in *Clostridiales* and *Lactobacillales* than infants fed non-MBM. Permanova also showed that bacterial compositions were different between males and females and between MBM and non-MBM feeding types. In conclusion, infant postnatal age, gender and feeding type significantly contribute to the dynamic development of the gut microbiome in preterm infants.

## Introduction

Over the past decade, advances in neonatal care have contributed to an increase in survival among very preterm infants [1, 2]. As a result, aspects of care management have shifted to focus on the prevention of diseases such as necrotizing enterocolitis (NEC) and sepsis, and improving long-term neurologic and developmental outcomes related to the immature neuro-immune systems and stressful early life experiences [3]. At the same time growing evidence supports that a functional communication exists between the central nervous system and gastrointestinal (GI) tract. In this brain-gut axis, the gut microbiome is proposed to play a key role in the early programming of health outcomes through a bidirectional signaling system, in terms of both top-down and bottom-up effects [4–8]. However, the developmental patterning of the gut microbiome and the factors affecting how colonization progresses in preterm infants during early life remains unclear and needs further investigation.

Development of the gut microbiome in neonates is complex and influenced by many factors, such as mode of delivery, infant gestational age and postnatal age, feeding types and nutrition, environmental factors, and antibiotics and/or probiotics usage [9]. Although the exact composition of a “normal” neonatal gut microbiome is still unknown, researchers have begun mapping the human GI microbiota using recently developed culture-independent, DNA-based genomic technologies [10]. After birth, newborn GI microbial colonization appears to begin with facultative anaerobes, followed by the establishment of anaerobic genera [9]. Compared to the adult, newborn GI microbiota may be more variable both over time (e.g. day-to-day) and between individuals [11]. Full-term infants born by vaginal delivery have greater microbial diversity that appears to be more desirable for both short and long term outcomes. Conversely, preterm infants born via caesarian section have been found to have less diversity within their microbiome because they are not exposed to maternal vaginal, fecal and epithelial microbes [12, 13].

Because of the various exposures often associated with admission to the neonatal intensive care unit (NICU), preterm infants have a delay in colonization with typical commensal bacteria. Rather, they are more likely to be colonized with potentially pathogenic microorganisms, showing reduced microbiota diversity, reduced levels of strict anaerobes, and a relatively high abundance of *Proteobacteria* [14–16]. Such alterations of gut microbial patterns in preterm infants have been found to correlate with life-threatening diseases such as NEC and late-onset sepsis [17, 18]. These alterations in GI microbiota, also called dysbiosis, are one of the pivotal factors linked to preterm infant mortality and morbidity [17–19]. However, the etiology of the unbalanced microbial profile in preterm infants’ GI tracts remains unclear and factors involved in their developmental progress warrant further investigation.

In the literature, feeding types have been found as one of the major factors influencing neonatal gut microbiome and GI function. For example, infants fed directly at the breast can gain additional variability in microbiota through breastmilk and also through contact with the mother’s skin [20]. Six *Bifidobacterium* strains have been isolated from human breastmilk showing phenotypical and genotypic characters of commercial probiotics, which are important specifically for neonates and for potential use in targeted interventions [21, 22]. In healthy full-term breastfed infants, human milk oligosaccharides can selectively shape the growth and function of beneficial GI microbiota. Full-term breastfed infants have more predominant *Bifidobacteria* and *Lactobacillus* in the GI tract compared to that of formula-fed infants [23]. In preterm infants, limited studies have shown very low amounts of anaerobes present in the GI tract, including *Bifidobacteria* and *Bacteroidetes*. This delay in anaerobic establishment is hypothesized to impede immune maturation in the preterm population [16, 24, 25]. The dynamics of bacterial colonization and composition of microbial communities in preterm infants remain largely unknown. Therefore, the effects of using mother’s own breastmilk feeding on infant gut microbiome development needs further investigation.

The objective of this prospective longitudinal study was to explore day-to-day gut microbiome patterns in preterm infants during their early life in the NICU, and to investigate the relationship between clinical factors (e.g. infant demographics, mode of delivery, feeding type, antibiotic use, and health conditions) and patterns of infant gut microbial colonization. By sequencing and analyzing the V4 region of the 16S rRNA genes of 378 fecal samples from 29 preterm infants born between 28 and 32 weeks gestation, we found dynamic developmental patterns of GI microbial colonization over the first 30 days of life which differed between male and female infants, and between infants fed by mother’s own breastmilk (MBM) and those fed human donor milk and/or formula (non-MBM).

## Materials and Methods

### Ethics Statement

The study protocol was approved by the institutional review boards (IRBs) of participating hospital and author affiliated university. All study procedures including clinical data collection and stool sample collection, storage and sequencing, and data analysis were approved by the IRBs.

### Patients and Samples

Subjects were recruited and samples were obtained from patients cared for at two Connecticut Children’s Medical Center (CCMC) NICU sites (Hartford and Farmington, CT, U.S.A.). Research assistants/nurses in the NICU identified eligible infants and obtained written informed consent from infants’ parents. All infants meeting the criteria and present in the participating NICUs during the study period were invited to participate. Inclusion criteria were stable preterm infants who were 28 0/7–32 6/7 weeks gestational age, 0–7 days old, and mothers were older than 18 years old to provide consent. Exclusion criteria were infants who had known congenital anomalies, severe periventricular/ intraventricular hemorrhage (≥ Grade III), undergone minor or major surgery procedures, or positive drug exposure history.

### Clinical Data Collection

Infant demographic and clinical characteristics including delivery mode, severity of illness, feeding type, and antibiotics use were collected. Severity of illness was measured by the Score for Neonatal Acute Physiology—Perinatal Extension-II (SNAPPE-II) [26]. Infant feeding types were collected daily over the first four weeks of life in the NICU and categorized as fed mother’s own breastmilk (MBM) and non-mother’s breastmilk (non-MBM) including human donor milk and/or formula. For this study, we chose the acronyms MBM and non-MBM to represent our groups because we clustered both donor milk and formula together for our analysis. We acknowledge that other researchers have used MoM (mother’s own milk) for their discussion of MBM.

Infant fecal samples were collected by trained bedside nurses in the NICU on a daily basis over the first four weeks of life, depending upon whether the infant had a stool. Stool samples from each infant were collected using sterile, disposable spatulas during diaper changes and then placed into a sterile specimen container. Samples were immediately frozen upon collection at -80°C, then transferred to the university laboratory and stored at -80°C until processing. All stool samples were assigned a unique study ID number and systematically entered into a specimen repository and database.

### DNA Extraction, sequencing, and sequence data processing

DNA was extracted from 0.25g of fecal sample using the MoBio Power Soil kit (MoBio Laboratories, Inc) according to the manufacturer instruction for the Eppendorf epMotion 5075 Vac liquid handling robot or manually. DNA extracts were quantified using a Syngery HT (Biotek, Winooski, VT) with the Quant-iT PicoGreen kit (Invitrogen, ThermoFisher Scientific). The V4 regions of the 16S rRNA gene was amplified with 515F and 806R primers containing Illumina adapters and golay indices on the 3’ end using 20 ng extracted DNA as template [27]. Samples were amplified in triplicate using Phusion High-Fidelity PCR master mix (New England BioLabs) with the addition of 10 μg BSA (New England BioLabs). The PCR reaction was incubated at 95°C for 3.5 minutes, the 30 cycles of 30 s at 95.0°C, 30 s at 50.0°C and 90 s at 72.0°C, followed by final extension as 72.0°C for 10 minutes. PCR products were quantified and visualized using the QIAxcel DNA Fast Analysis (Qiagen). PCR products were normalized based on the concentration of DNA in the 350–400 bp region and pooled using the QIAgility liquid handling robot (Qiagen). Pooled PCR products were cleaned using the Gene Read Size Selection kit (Qiagen) according to the manufacturer’s protocol. The cleaned pool was sequenced on the MiSeq using v2 2x250 base pair kit (Illumina, Inc).

The sequences were demultiplexed requiring 0 mismatches in the index sequences and Q25 minimum, merged using SeqPrep and filtered for length (maximum 300bp) using a custom script (https://github.com/mcnelsonphd/16S-RDS/blob/master/Qiime_Process) [28]. Using QIIME (Quantitative Insights Into Microbial Ecology) software, operational taxonomic units (OTUs) were determined by clustering reads to the Greengenes reference 16S reference dataset (2013–08 release) at a 97% identity, and then performing *de novo* OTU clustering on reads that failed to cluster to a reference [28, 29]. The dataset was filtered to remove OTUs present at less than 0.0005% [28, 30]. The data was rarified to 10,000 reads per sample.

### Statistical Analysis

Clinical data, OTU tables and the Gini–Simpson α-diversity index calculated from QIIME process were imported into R 3.2.0 for statistical analysis. Exploratory data analyses including scatter plot, multidimensional scaling (MDS), and taxonomy graph techniques were conducted to display the a-diversity (Gini–Simpson diversity index), β-diversity, and the composition of organism in the preterm infants’ gut-microbiome community. Generalized linear mixed models (GLMM) were used to analyze the association between demographic characteristics and clinical variables with α-diversity. β-diversity was analyzed using both Bray-Curtis and Jaccard in Vegan package (https://cran.r-project.org/web/packages/vegan/index.html) and visualized using multidimensional scaling (MDS). To further examine the effect of demographic and clinical characteristics on gut microbiome patterns, the permutational multivariate analysis of variance using distance matrices (PERMANOVA) and multivariate analogue of Levene's test for homogeneity of groups dispersions (BETADISPER) were conducted. Indicator value analyses using Indicspecies package (https://cran.r-project.org/web/packages/indicspecies/index.html) were also performed to identify species that drive the differences in microbiome patterns among preterm infants with different clinical characteristics.

## Results

A total of 30 preterm infants were enrolled in the study from January, 2014 to January 2015. One infant’s stool was not included in the analysis due to a technical freezer related issue. In the final analysis of 29 subjects (Table 1), the majority of the infants were male (52%), white (75%), non-Hispanic (69%), and delivered by cesarean-section (55%). Infants were born at 31.3 ± 1.7 weeks of gestation and with 1459.8 ± 445.3 g of birth weight. Enteral feedings were introduced between postnatal day 1 to day 6 (2.1 ± 1.0 days), depending on the infants’ clinical condition. During the study period, 69.0% of the total feedings were with MBM and the days of using MBM for each infant varied from 0 to 20 days (6.0 ± 5.9 days). The infant feeding type was then categorized based on the MBM fed proportion of all feedings during the study period that the infant was included in the MBM group when he/she received MBM in 50% or more of feedings, otherwise the infant was included in the non-MBM group. The number of NPO days were similar between the MBM and non-MBM groups. Feeding types received by male and female infants were also similar during the study period (Table 1). In this group of infants, the time to regaining birth weight was from 4 to 18 postnatal days (11 ± 4 days).

**Table 1:** Demographic and clinical characteristics of the preterm infants.

Demographic		n	Percent (%)
Gender	Male	15	51.7
	Female	14	48.3
Race	White	22	75.9
	African American	5	17.2
	Asian	1	3.4
	Not known	1	3.4
Ethnicity	Hispanic	9	31.0
	Non-Hispanic	20	69.0
Delivery type	Vaginal	13	44.8
	Cesarean section	16	55.2
PROM	Yes	13	44.8
	No	16	55.2
Birth	Multiple birth	12	41.4
	Single birth	17	58.6
Resuscitation at birth	Yes	18	62.1
	No	11	37.9
Antibiotic Use	First 48 hours	20	69.0
Feeding types (by gender)	MBM^A^: Male	10	34.5
	Female	10	34.5
	Total	20	69.0
	Non-MBM: Male	5	17.2
	Female	4	13.8
	Total	9	31.0
	**Mean (SD)**	**Range**	
Gestational age (wks)	31.3 (1.7)	28.1–32.4	
Birth weight (g)	1459.8 (445.3)	703–2640	
Birth length (cm)	40.0 (4.3)	32.5–50.5	
Birth head circumference (cm)	28.1 (2.5)	24.0–34.5	
SNAPEII	8.6 (10.5)	0–31	

Note: PROM = premature rupture of membranes

^A^MBM = mother’s own breastmilk feeding

Non-MBM = non-mother’s own breastmilk feeding including human donor’s milk and formula feeding; SNAPEII = Neonatal Acute Physiology–Perinatal Extension-II.

A total of 378 stool samples were collected from 29 preterm infants during their first 30 days of life. The average number of stool collections for each infant was 13 ± 4.8 with a median of 14 collections per infant and minimum of 1 sample per infant. The mean interval between sample collections was 1.5 ± 1.1 days with a median of 1 day. Twenty-eight out of the 378 samples including 10 meconium stools yielded DNA concentration less than 1ng/ml and were therefore excluded from Miseq sequencing due to the low concentrations. Meconium, a sticky stool with a color of dark olive green, is the earliest stool of infants in the first week of life. In total, 25.6 million high quality and chimera free reads were produced. Twelve samples yielded less than 10,000 reads and were excluded for QIIME analysis. There was only one sample collected from postnatal day 4 that passed the 10,000 cutoff and was removed from the analysis to reduce bias.

### Microbiome community patterns in preterm infants

**Fig 1:**
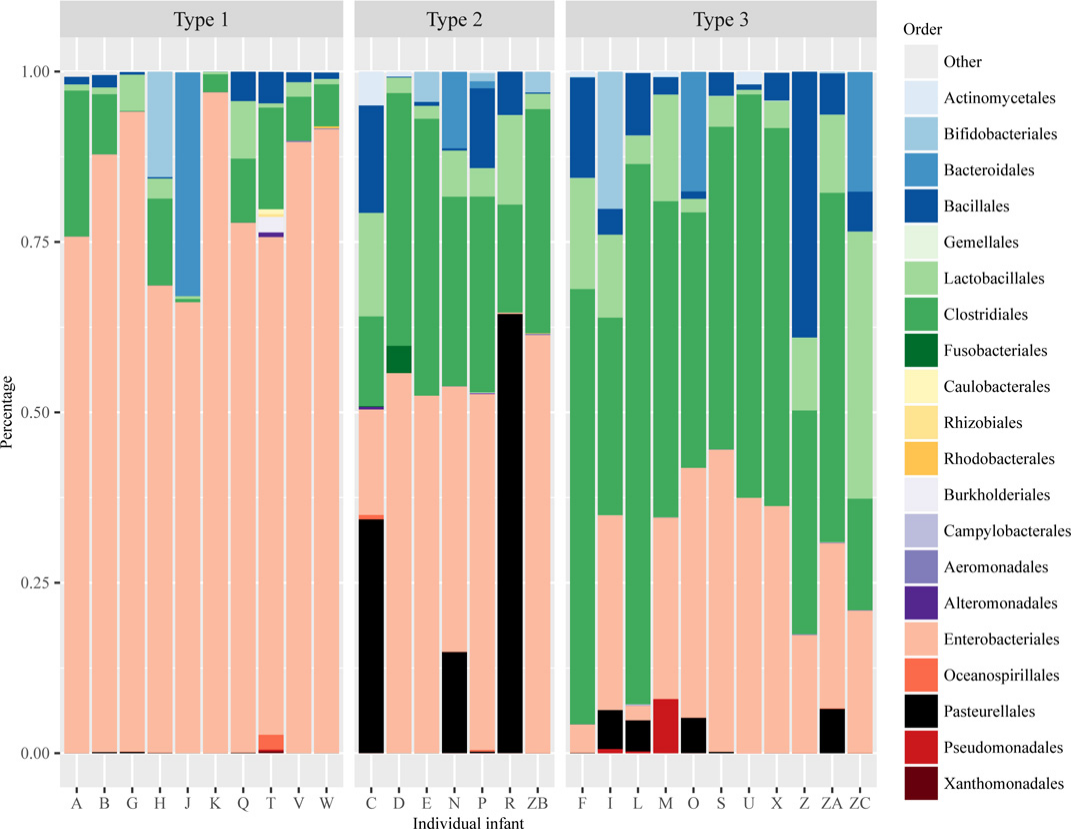
Distribution of mean relative abundance of taxa per infant. Each of the above stacked bar plots illustrates the average relative abundance (y-axis) of the most abundant gut microbiota at the order level. X-axis is a summary of all samples from each infant. Fig 1 displays three types of gut microbiome community pattern: Type 1 = *Enterobacteriales* dominated (n = 10); Type 2 = mixed pattern (n = 7); Type 3 = *Clostridiales* and *Lactobacillales* dominated (n = 11). One infant was not included in Fig 1 because only 1 sample was available from this infant, which may bias the pattern.

**Fig 2:**
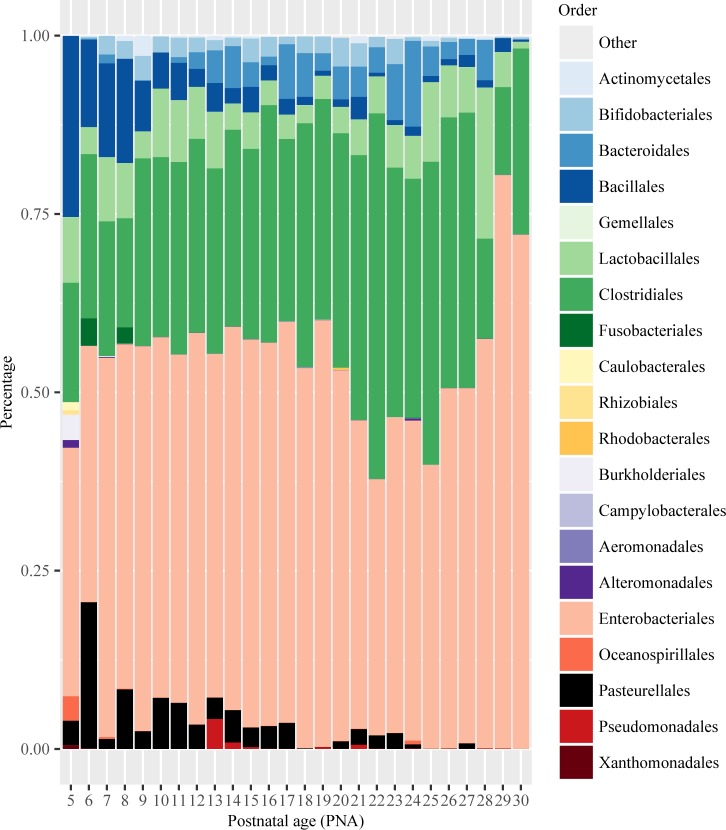
Distribution of mean relative abundance of taxa for temporal (daily) development of taxa during the first 30 days of life. Each of the above stacked bar plots illustrates the average relative abundance (y-axis) of the most abundant gut microbiota at the order level from each infant. X-axis is a summary of all samples for each postnatal day.

The data yielded sequences belonging 5 phyla, 11 classes, 42 families and 64 genera. *Proteobacteria* was the most abundant phylum, accounting for 54.3% of the total reads, followed by *Firmicutes* 39.2%, *Bacteroidetes* 3.9%, *Actinobacteria* 2.4% and Fusobacteria 0.2%. The distribution of the mean relative abundance of OTUs at order level were calculated for each infant (Fig 1) and at each day (Fig 2) during the first 30 days of life. Gut microbiome communities in this group of preterm infants appeared individually diverse and often dominated by a single OTU. Three emerging types of composition patterns were found, Type 1 with Enterobacteriales dominating, Type 2 with mixed composition, and Type 3 with Clostridiales and Lactobacillales dominating (Fig 1). Comparisons of infant demographic and clinical characteristics among the three types of microbial composition are shown in S1 Table and examples of daily gut microbiome development from individual infants that belong to three types of microbiome patterns are shown in S1 Fig. A heat map of daily microbial development was created at genus level and showed a shifting pattern of increasing *Clostridium* and *Bacteroides*, and decreasing *Staphylococcus* and *Haemophilus* over time during early life (see S2 Fig). The nonmetric multidiminsional scaling (NMDS) of the samples based on Bray-Curtis dissimilarity of the OTUs was calculated and displayed (S3 Fig).

Biodiversity of the gut microbiome was measured using Gini-Simpson α-diversity index in this study, which provides quantitative measures of the richness and evenness of the species in a microbiome community. Richness measures the number of species present in a sample and evenness is a measure of the relative abundance of the different species composite in the sample. A community dominated by only a couple of species is less diverse than one in which several different species have relatively similar abundance. The α-diversity ranges from 0, the lowest diversity to 1, the highest diversity. The gut microbiome data from our study showed that α-diversity increased from 0.31 ± 0.32 at postnatal day 5 of life to 0.49 ± 0.19 at day 30 of life, with an average increase of 0.008 per day, p < 0.001. To further explore contributing factors to the microbial complexity among infants, generalized linear mixed models (GLMM) were performed to assess the effect of different infant characteristics on the richness and evenness of the infant gut microbiome with α-diversity. The contributing factors entered to the models included gender, premature rupture of membranes, birth gestational age, delivery mode, severity of illness, feeding type, antibiotics use, location (two NICU sites), and postnatal days. Results showed that time (postnatal days), p < 0.01, feeding type (using MBM or not), p < 0.01, and gender, p < 0.05 were significantly associated with α-diversity.

### Different Microbiome patterns in Male and Female Infants

Gender was found to be a significant driving factor in the development and maturation of gut microbiota in the mixed-effects models. Prior to comparing gender differences in microbiome community, we tested whether the number of males and females was equal among clinical factors that may be associated with microbiome development, such as delivery mode, PROM, antibiotic use, and feeding type. Crosstabs and Chi-squares analyses were conducted and results showed no significant differences between the two genders in these clinical factors (S4 Fig). Days of antibiotic use were also examined using independent t-test and shown to be similar between the males (1.67 ± 2.26 days) and females (2.46 ± 2.96 days), p > 0.05.

**Fig 3:**
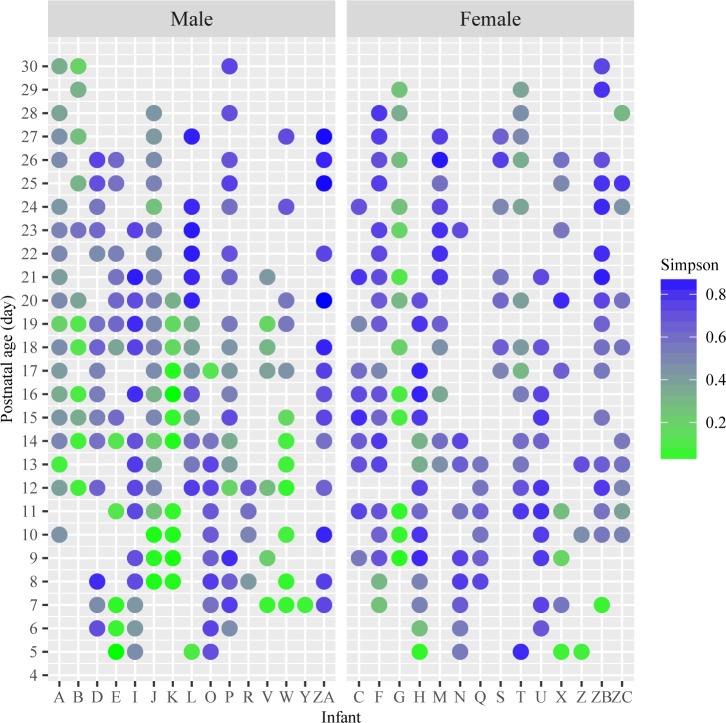
Gini-Simpson α-diversity index between male and female infants. X-axis represents the individual infant. Y-axis represents the postnatal age after birth, samples from postnatal day 1 at the bottom and the sample on postnatal day 30 at the top. The value of Gini-Simpson α-diversity index for each stool sample was illustrated by the color gradient from green as the lowest and the dark blue as the highest. Left figure shows males and right figure shows female data.

To examine the difference in the bacterial community between male and female infants, we illustrated the differences in α-diversity between the genders using scatter plot (Fig 3). Male infants began with a low diversity index (0.34 ± 0.30), whereas female infants tended to have a higher diversity index (0.49 ± 0.23) shortly after birth compared to males during the first 10-days of life. Then, the diversity of gut microbial community developed over time and appeared similar in males (0.61 ± 0.18) and females (0.69 ± 0.17) during the third 10-days of life. The overall mean of α-diversity was higher in females (0.58 ± 0.22) then males (0.48 ± 0.26) during first 30 days of life, p < 0.05.

The PERMANOVA and BETADISPER analyses were performed to test whether the bacterial community structure differed between genders. The PERMANOVA partitions the variability between factors based on Bray-Curtis dissimilarity matrix. Results of the testing indicated statistically significant differences in gut microbiome community between different genders (F = 32.22, R squared = 0.08, p < 0.001). The BETADISPER calculates the average distance of group members to the group centroid or spatial median in a multivariate space. The average distance to median from two groups differed by 9% (Male: 0.47; Female: 0.56).

**Fig 4:**
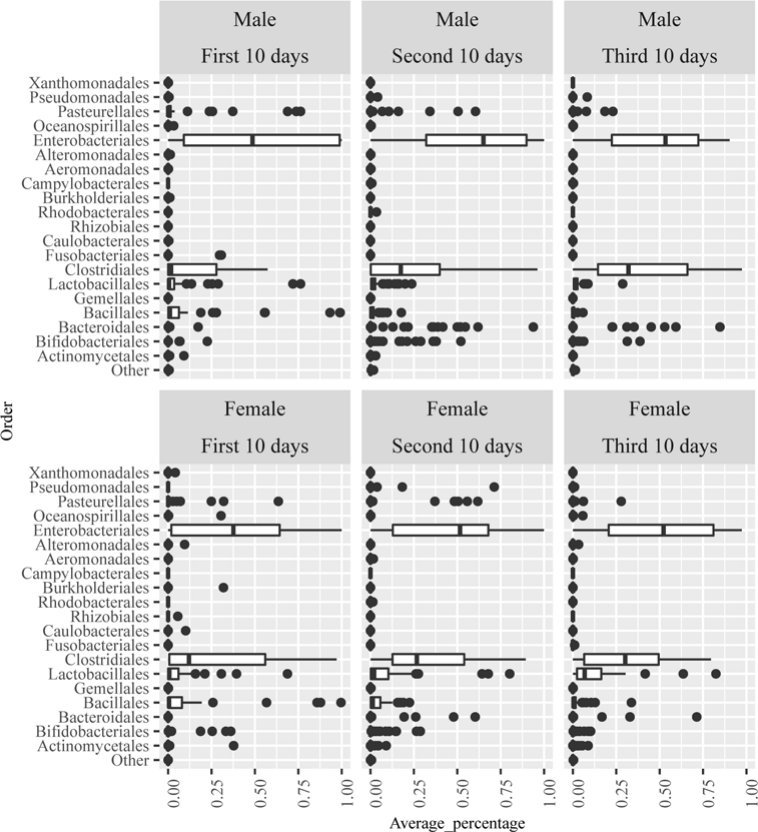
Abundance variation boxplots for the top abundant OTU at the order level in males and females during the first, second and third 10-days periods of life. Upper half of the figure shows the male data and lower half of the figure shows female data.

Different gut microbiome patterns (Fig 1) were found between genders. Males were more likely to belong to Type 1 pattern with *Enterobacteriales* dominated, but less likely to belong to Type 3 pattern with *Clostridiales* and *Lactobacillales* dominated, while females were the opposite (Type 1: male = 6, female = 4; Type 2: male = 4, female = 3; Type 3: male = 4, female = 7). Furthermore, the variation of the abundance at the order level in the male and female infants during the first, second and third 10-days of life (postnatal days 5–10; 11–20; and 21–30) were illustrated as box-and-whisker plots in Fig 4. Female infants showed a pattern of higher abundance at the order level in *Clostridiates*, and less abundance in *Enterobacteriales* than males during their first 10-day periods of life. Indicator species analysis was then performed to identify the genera that reflected the differences in gut microbiota among preterm infants between genders (Table 2). Among all the microbes at the genus level, the abundance of *Pantoea* and *Campylobacter* (in *Proteobacteria* phylum) were statistically higher in males than in females, while the abundance of unidentified microbes and *Yersinia* (in *Formicate* and *Proteobacteria* phyla) are significantly higher in females than males from birth to 30 days of life (Table 2).

**Table 2:** Different indicator species of gut microbiome between genders.

Phylum	Order / Genus	Indicator Value
Male		
Proteobacteria	Enterobacteriales / *Pantoea*	0.60**
Proteobacteria	Campylobacterales / *Campylobacter*	0.29*
**Female**		
Firmicutes	Clostridiales / other	0.81**
Firmicutes	Other / other	0.69**
Firmicutes	Clostridiales / other	0.58**
Proteobacteria	Enterobacteriales / *Yersinia*	0.51**

** p < 0.01

* p < 0.05

### Different Microbiome Patterns by Feeding Types

Feeding type was found to be another significant driving factor associated with microbiome diversity in the mixed-effects models. In order to further check whether the two feeding types (MBM and non-MBM) were equally distributed among other clinical factors that may be associated with microbiome development, such as delivery mode, PROM, and antibiotic use, we conducted crosstabs and Chi-squares analyses. Results showed that there were no significant differences in these clinical factors between the two feeding types (S5 Fig). Days of antibiotic use were also found to be not significantly different between the MBM (2.37 ± 3.04 days) and non-MBM fed (1.33 ± 1.00 days) infants, p > 0.05.

**Fig 5:**
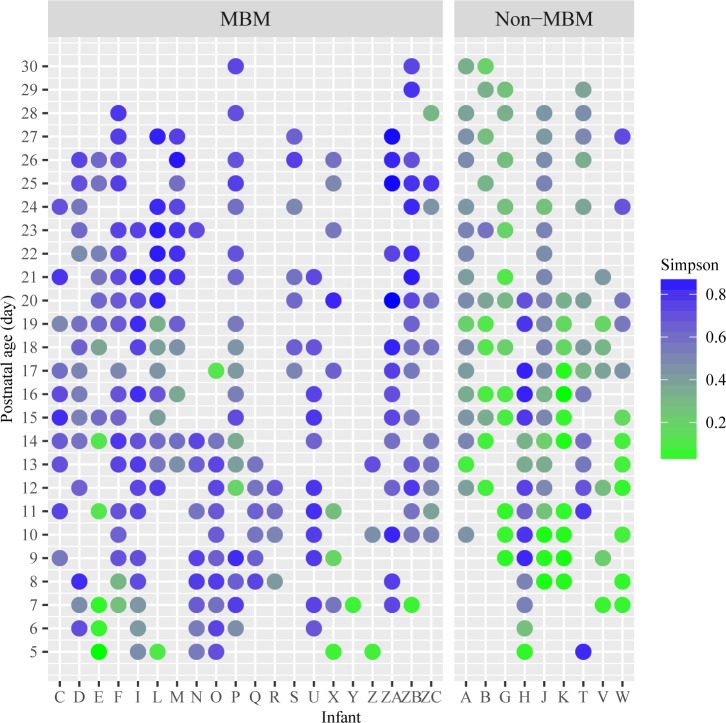
Gini-Simpson α-diversity index of microbiome community between infants with different feeding type from postnatal day 5 to 30. MBM = mother’s breastmilk feeding; Non-MBM = non-mother’s breastmilk feeding including human donor’s milk and formula. X-axis represents the individual infant. Y-axis represents the postnatal age after birth, samples from postnatal day 1 at the bottom and the sample on postnatal day 30 at the top. The value of Gini-Simpson α-diversity index for each stool sample is illustrated by the color gradient from green as the lowest and the dark blue as the highest. Left figure shows MBM and right figure shows non-MBM feeding data.

When examining the relationship of feeding types and infant gut microbiome, MBM feeding was found to be associated with higher diversity of infants’ gut microbiome compared to non-MBM feeding, including human donor’s milk and/or formula. On average, α-diversity of the gut community from infants who were fed by MBM (0.68 ± 0.18) tended to be higher than infants who did not receive MBM (0.42 ± 0.26), p < 0.001. The infants fed by non-MBM tended to have less complex community at the first sampling points and the increasing rate of diversity was slow over time. The differences between feeding types on α-diversity were also illustrated using scatter plot (Fig 5).

To further investigate the difference in the bacterial community between different feeding type, PERMONOVA and BETADISPER tests based on Bray-Curtis dissimilarity matrix were conducted to examine the differences within community structures. The result showed significant difference between MBM fed infants and their cohort at the genus level (F = 36.84, R squared = 0.10, p < 0.001). BETADISPER showed that the average distance to median among MBM fed infants was 0.56, which differed from the infants with non-MBM feeding, 0.43, by 13%.

**Fig 6:**
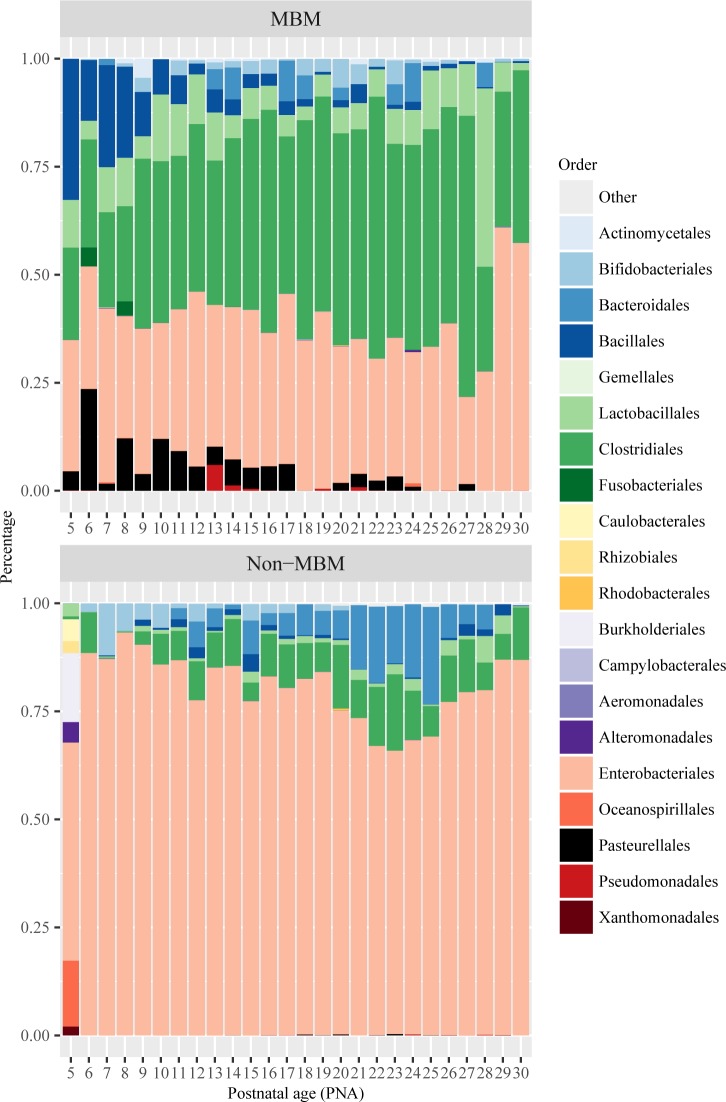
Distribution of relative abundance of taxa between feeding types during the first 30 days of life. MBM = mother’s breastmilk feeding; Non-MBM = non-mother’s breastmilk feeding including human donor’s milk and formula. Each of the above stacked bar plots illustrates the average relative abundance (y-axis) of the most abundant gut microbiota at the order levels. X-axis represents postnatal day.

Gut microbiome community patterns (Fig 1) were found to be largely different between MBM and non-MBM fed infants during early life. The MBM fed infants were more likely to belong to Type 2 pattern with mixed compositions and Type 3 pattern with *Clostridiales* and *Lactobacillales* dominating. The non-MBM fed infants were more likely to belong to Type 1 pattern with *Enterobacteriales* dominated, (Type 1: MBM = 2, non-MBM = 8; Type 2: MBM = 7, non-MBM = 0; Type 3: MBM = 10, non-MBM = 1). MBM fed infants had significantly higher abundance in *Clostridiales* (p < 0.001) and *Lactobacillales* (p < 0.05) than non-MBM fed infants. Furthermore, we compared the composition of bacterial community by the feeding types during each postnatal day (Fig 6). In the MBM fed infants, the composition of *Enterobacteriales* shifted from about 50% at postnatal day 5–20 down to about 30% thereafter, whereas, *Clostridiales* and *Lactobacillales* developed quickly from about 20–30% at postnatal day 5–7 up to 60–70% after day 20 (Fig 6). On the contrary, non-MBM fed infants had a different gut microbiome community pattern that was dominated by *Enterobacteriales* (more than 65%) throughout the first 30 days of life and the pattern shifted slowly day-by-day (Fig 6). The indicator species analysis was then conducted at the genus level to identify the differences of the bacterial composition between feeding types. Results showed that *Granulicatella*, *Lactobacillus*, and other genera belong to *Firmicutes* phylum were statistically higher in the group of MBM fed infants than their cohorts (Table 3).

**Table 3:** Different indicator species of gut microbiome between feeding types.

Phylum	Order / Genus	Indicator Value
**MBM Feeding**		
Firmicutes	Clostridiales / other	0.76**
Firmicutes	Lactobacillales / other	0.71**
Firmicutes	Other / Other	0.70**
Firmicutes	Lactobacillales / *Granulicatella*	0.68**
Firmicutes	Clostridiales / other	0.53**
Firmicutes	Lactobacillales / *Lactobacillus*	0.51**
**Non-MBM Feeding**		
Bacteroidetes	Bacteroidales / Prevotella	0.20*

** p < 0.01

* p < 0.05

MBM = mother’s breastmilk feeding

## Discussion

In this study, preterm infants’ gut microbiome patterns varied substantially among individual infants (Fig 1 and S2 Fig) and *Proteobacteria* was found to be the most abundant phylum. We also found that over the first 30 days of early life, gut microbiome diversity index began low and increased daily after birth for this group of preterm infants. The gut microbiome community patterns shifted by increases in *Clostridium* and *Bacteroides* sequences, and decreases in *Staphylococcus* and *Haemophilus* with time (S1 Fig). Meanwhile, time (postnatal days of life), gender, and mothers’ own breastmilk feeding were found to be significant driving factors influencing the dynamics of gut microbiome development in preterm infants. Our findings are consistent with previous studies that demonstrate preterm infants, as opposed to healthy full-term infants, show a remarkably less diverse microbiome, relatively low abundance *Bifidobacteria* and *Bacteroidetes* and high abundance of *Proteobacteria*, and increased colonization by pathogens [15, 16, 24, 31, 32]. Healthy newborn gut microbial community begins with colonization of facultative anaerobes, followed by the establishment of anaerobic genera, such as *Bifidobacterium*, *Bacteroides*, and *Clostridium* [33]. An appropriate balance in the diversity and type of microbes within the microbiome community plays a critical role in promoting immune maturation and maintaining the host health status in this population. The alteration or imbalance in the richness and diversity of the microbiota termed as *dysbiosis*, has also been recently attributed to delayed maturation of the microbiota in infants [34]. Distortions and/or delayed maturation of the gut microbiota with community changes dominated by *Proteobacteria* and *Firmicutes* have been found to be correlated with NEC and late-onset sepsis in preterm infants [17–19, 35]. A recent study also shows that reduced bacterial diversity and the absence of certain bacterial classes of the gut, such as *Clostridia* in stool samples may be associated with the severity of NEC [36].

The mechanistic interplay of gut microbiome development and early life environmental factors are still poorly understood. Dysbiosis in high risk infants is associated with exposure to potentially detrimental factors, including caesarean delivery, increased exposure to antibiotics and acid-suppressing agents, lack of breast-feeding, and exposure to environmental toxicants [35]. Moreover, exposure to early life physiological and psychosocial stressors, such as prolonged painful experience and maternal-infant separation, can lead to oxidative stress within the intestines, which may modulate the process of microbiome establishment in premature infants [37, 38]. More research is needed to better understand the mechanism of gut microbiome development in high risk including preterm infants.

### Gender and gut microbiome

We found that gut microbiome communities in males and females were different after birth in this group of preterm infants. Male infants began with a lower diversity index, whereas females tended to have a higher diversity shortly after birth. Interestingly, our results show that female infants were more likely to have higher abundance of *Clostridiates*, and less abundance of *Enterobacteriales* than males during their early life. As these differences in the composition of the microbiota correlate with different clinical outcomes, it will be important to confirm these findings in studies at other sites and with an even larger number of subjects. To the best of our knowledge, this is the first study that reported gender associations of the gut microbiome composition in human neonates. Studies reporting gender specific differences in gut microbiome are few and inconsistent in human. One study reported strong associations between gender and microbial community types identified at several body sides in young adults (median: 25 years old) and showed that men more likely harbor fewer *Bacteroides* and higher *Prevotella* in the stool community compared to women [39]. Whereas, a recent study in older adults showed a lower abundance of *Bacteroidetes* phylum in women than men (58 ± 13 years old) [40]. However, gender differences in initial gut microbiota colonization and abundance of select taxa of early life in neonates have not been documented. One report briefly mentioned that males had a lower relative abundance of *Bacteroides* species than females in Caucasian infants at 3 months of age [41]. The mechanism of the gender specific differences on gut microbiome community remains poorly understood.

It has been speculated that the variation of gut microbiome communities influenced by sex are related to hormone-immune-microbe interactions and genetic traits [42]. Evidence has been established that many autoimmune disorders are gender related, generally more common in females, while gut microbiome is involved. Sex steroids may play an important role of shaping the gut microbiome in a sex specific way after puberty, such as in dietary and autoimmune disease studies [40, 43]. However, this mechanism may not be appropriate to explain the gender differences found in our infant gut microbiome study. Animal studies recently demonstrated that males and females respond to diet and pathologic and probiotic microorganisms differently [44, 45]. Differences in relative abundance of microbial species were found in the female GI tract of mice as compared to the male [44], which seems consistent with our study results where we detected differences in the microbiome of female and male infants. Additionally, cytokine productions were observed to be significantly different between male and female rodents in the colon, cecum and liver at basal levels and also at experimental conditions, indicating sex traits and sex related host immune-microbe responses to newly introduced diet and microorganisms [44, 45]. Meanwhile, colon tissues of male and female mice were also found to be significantly different in that males had higher concentrations of short-chain fatty acids (butyrate and acetate) and females had greater increases in o-phosphocholine or histidine [44]. These recent findings suggest that sex biases in the fecal communities may be influenced by sex specific metabolic and immune activities, as well as immunoregulation in GI tissues. However, the mechanisms underlying and gender differences in gut microbiome community in early life including host immune functions and metabolic environment in the GI are still largely unknown and further investigations are needed.

### Feeding type and gut microbiome

We found that infants fed MBM had a significantly more diverse gut microbiome and higher abundance of *Clostridiales* and *Lactobacillale* than those fed non-MBM (human donor milk and/or formula). In contrast, infants fed non-MBM had a different type of microbiome community that was extensively dominated by *Enterobacteriales* throughout the first 30 days of early life. Interestingly, we found these difference even though the infants in the MBM group could have received some donor milk or formula during the study. It appears that receiving greater than 50% of MBM matters. These differences could be attributable to a reduced number of bacteria in pasteurized donor milk and formula or differences in the composition in mother’s milk, the donor milk, and formula [46]. Our data also suggest that the microbiome from mother’s breastmilk potentially play important roles in infant health. Recent evidence supports that mother’s own breastmilk feeding promotes the growth of commensal bacteria in the infant GI tract [23, 25, 46–48]. The composition of infant diet including sugars, amino-sugars, and fatty acids has shown an important role in defining gut microbiota in immature infant GI tracts [48]. One of the mechanisms of the effect of breastmilk on shaping infant gut microbiome is that breastmilk provides many nutrients for supporting the growth of bacteria that are adapted to the infants digestive tract and typically do not cause disease. Human milk oligosaccharides (HMOs), prebiotics, the third largest component of breastmilk are shown to have bio-active effects beyond providing nutrition to the infant. Specific beneficial microbes including *Bifidobacteria* and *Bacteroidetes* may grow quickly by digesting and utilizing HMOs through specific glycosidases, but most potentially pathogenic microbes, such as *Enterobacteriaceae* are unable to utilize HMOs as a food source because of lack of specific enzymes [49]. Therefore, healthy breastmilk fed full-term infants have *Bifidobacteria* and *Bacteroidetes* dominant in their gut microbiome community and have nearly twice the abundance of gut bacterial cells than non-breastmilk fed infants [47].

Currently, little is known about the influence of feeding type on the development of gut microbiota in preterm infants. Breastmilk produced by mothers of preterm infants has been found to be largely different from that of mothers of full-term infants including the individual variations and the amounts and types of HMOs, as well the “premature” human milk may impact the premature gut in different mechanisms [50]. Preterm infants may also respond differently to HMOs compared to full-term infants. One study showed that supplemental HMOs administrated to the feedings in preterm infants did not show the expected increase in abundance of *Bifidobacteria* [25]. Our result appears consistent with previous studies that preterm infants did not have a significant increase in *Bifidobacteria* fed by MBM, but had higher abundance of a group of *Firmicutes* bacteria (e.g. *Clostridiales* and *Lactobacillales*) in MBM fed infants than non-MBM fed infants. Compared to term infants, investigation of preterm infant gut microbiome and associated factors contributing to health is challenging because of the complex situations of during their early life. In addition to the factor of feeding type, we also need to consider that the NICU environment, including feeding tubes, delayed enteral feeding, medication use and other hospital-acquired organisms contributes to the microbiome development in preterm infants. In addition, these infants have a reduced exposure to microorganisms from their parents, siblings, pets and the environment.

In summary, using prospective longitudinal method and data from multiple time points we found unique gut microbiome patterns and developmental trends over time during early life of premature infants. While, this is the first study to date that reported gender differences in gut microbiome of preterm infants, one of its limitations remains a small sample size. Another limitation is that only stable preterm infants without severe comorbidities, i.e., NEC or late onset sepsis were recruited in this study and it may limit the generalizability of these results to high-risk preterm population. Future studies with larger sample sizes and multiple geographic locations, as well as including clinically unstable infants are needed to lead a greater understanding of the concept of dysbiosis and fully describe the associations of contributing factors including gender and feeding type with microbiome and health outcomes in premature infants.

## Supporting Information

S1 Fig Examples of daily gut microbiome development from individual infants. Infant A belongs to Type 1 with *Enterobacteriales* dominated; Infant P belongs Type2 with mixed pattern; and Infant F belongs to Type 3 with *Clostridiales* and *Lactobacillales* dominated.
(TIFF)

S2 Fig Distribution of Bacterial genera by postnatal age. This heat map shows the abundance (shades of blue) of detected genera in columns aggregated by postnatal age (rows). The values are the sum of the number of reads. The phyla level membership of each genera is indicated by the color bar along the top of the heat map. The genera have been clustered by distribution similarity.
(PDF)

S3 Fig Nonmetric multidimensional scaling (NMDS) of the samples based on Bray-Curtis dissimilarity of the OTUs. All samples belonging to an infant have the same color and shape combination. This demonstrates the similarity of the communities within an individual.
(TIFF)

S4 Fig Comparison of gender with clinical factors. C-section = Cesarean section, PROM = premature rupture of membrane; MBM = mother’s breastmilk.
(DOCX)

S5 Fig Comparison of feeding type with other factors. C-section = Cesarean section, PROM = premature rupture of membrane; MBM = mother’s breastmilk. There were no significant differences between the MBM and non-MBM feeding in these factors.
(DOCX)

S1 Table Infant Demographic and Clinical Characteristics among the Three Types of Gut Microbial Composition (DOCX)

S1 File Data sharing files. (ZIP)
