# Supplementary figures and images for: Gut Microbiome Developmental Patterns in Early Life of Preterm Infants: Impacts of Feeding and Gender
Source: PLoS One. 2016 Apr 25;11(4):e0152751. doi: 10.1371/journal.pone.0152751 (PMC4844123; doi:10.1371/journal.pone.0152751)

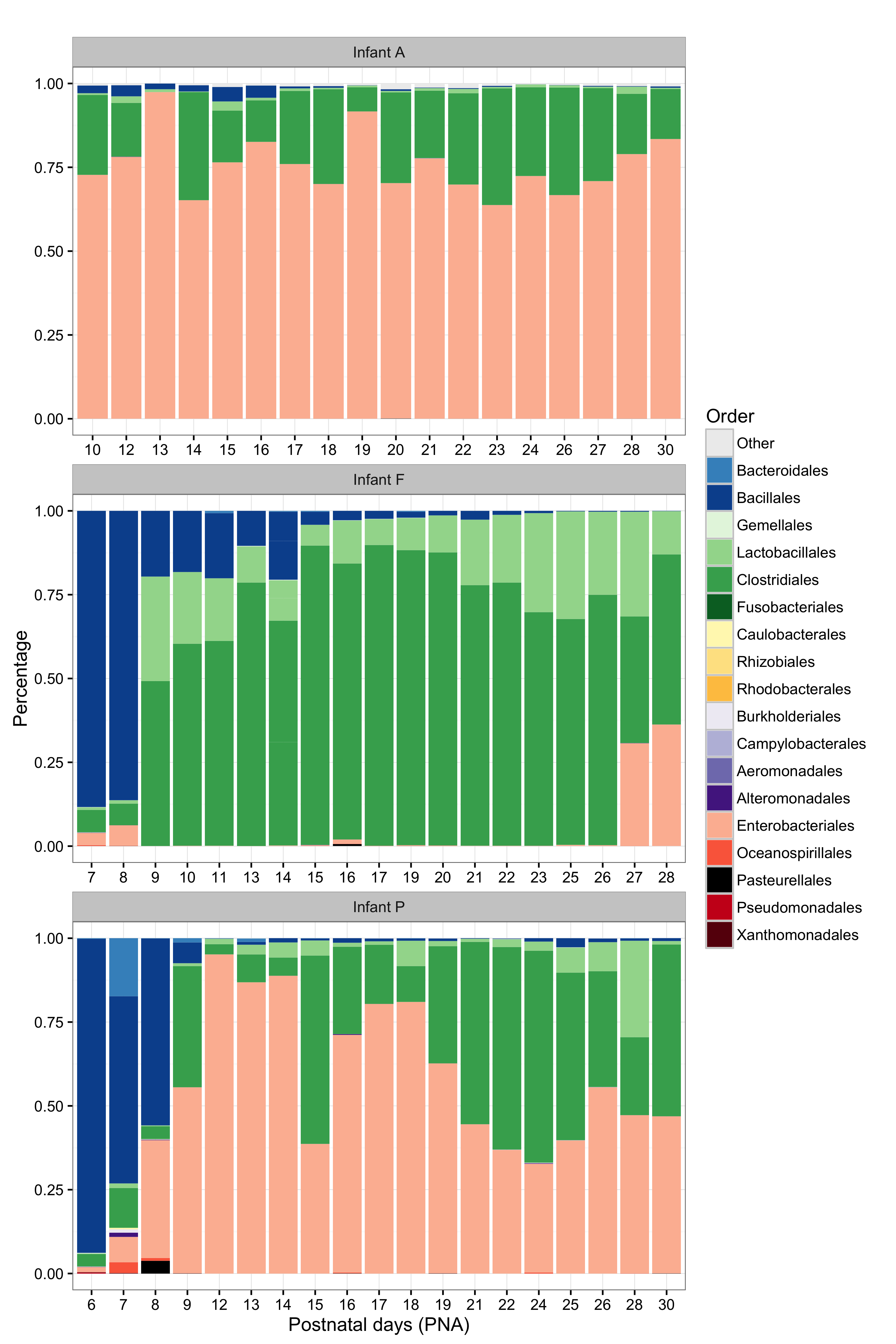

Supplement: S1 Fig — Infant A belongs to Type 1 with Enterobacteriales dominated; Infant P belongs Type2 with mixed pattern; and Infant F belongs to Type 3 with Clostridiales and Lactobacillales dominated. (TIFF) [file pone.0152751.s001.tiff]

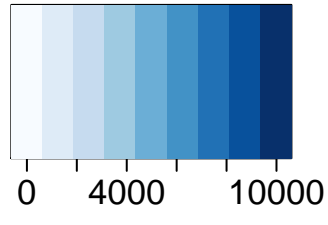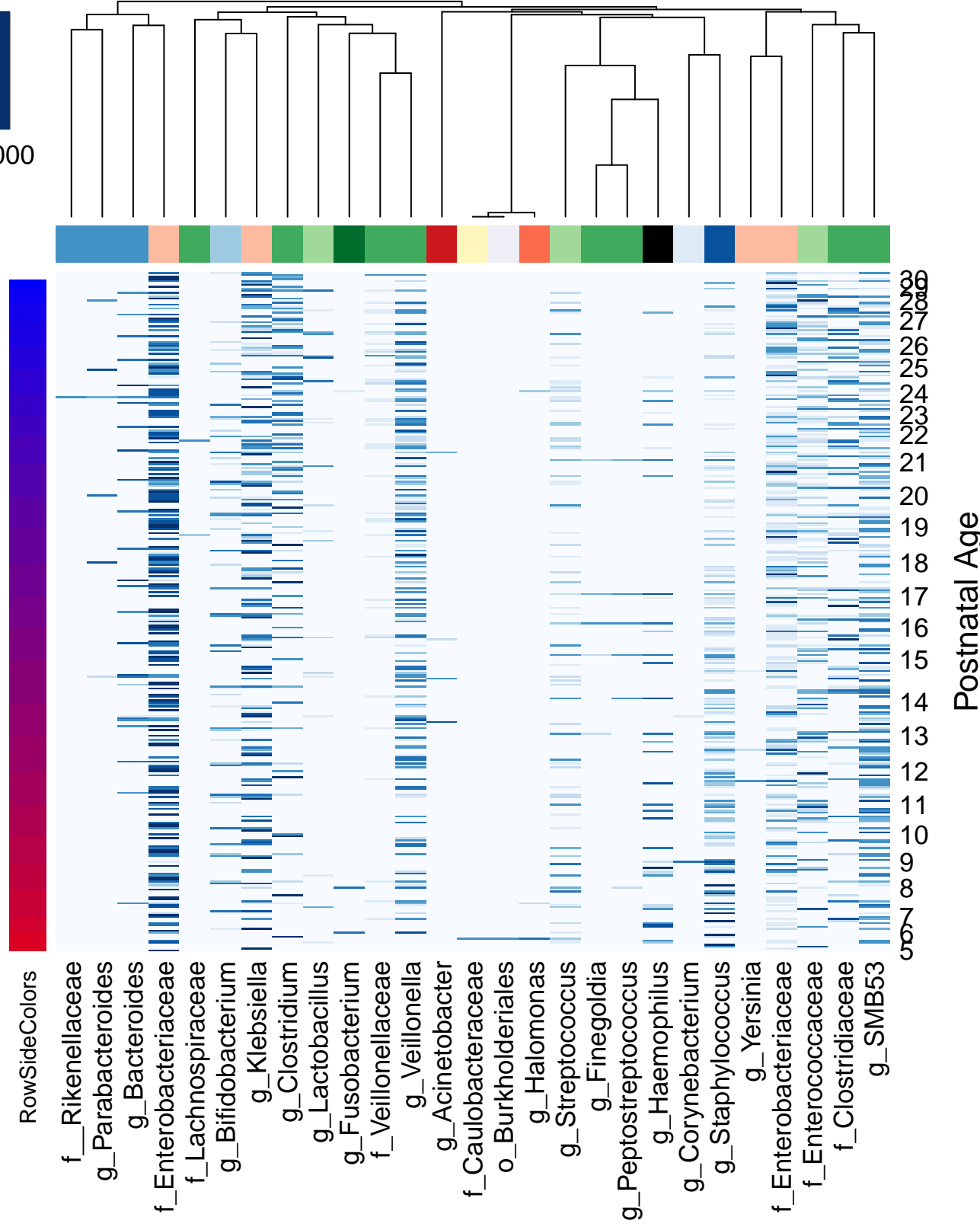

Supplement: S2 Fig — This heat map shows the abundance (shades of blue) of detected genera in columns aggregated by postnatal age (rows). The values are the sum of the number of reads. The phyla level membership of each genera is indicated by the color bar along the top of the heat map. The genera have been clustered by distribution similarity. (PDF) [file pone.0152751.s002.pdf]
