# Supplementary material for: Gut Microbiome Developmental Patterns in Early Life of Preterm Infants: Impacts of Feeding and Gender
Source: PLoS One. 2016 Apr 25;11(4):e0152751. doi: 10.1371/journal.pone.0152751 (PMC4844123; doi:10.1371/journal.pone.0152751)
Supplement: S1 Table — (DOCX) [file pone.0152751.s006.docx]

| **Microbial Composition** | | **Type 1 (n=10)** | **Type 2 (n=7)** | **Type 3 (n=11)** |
| --- | --- | --- | --- | --- |
|  |  | Frequency (%) | Frequency (%) | Frequency (%) |
| **Gender** | **Male** | 6 (60%) | 4 (57.1%) | 4 (36.4%) |
|  | **Female** | 4 (40%) | 3 (42.9%) | 7 (63.6%) |
| **Feeding type** | **MBM** | 2 (20%) | 7 (100%) | 10 (90.9%) |
|  | **Non-MBM** | 8 (80%) | 0 | 1 (0.1%) |
| **Delivery type** | **Vaginal** | 4 (40%) | 5 (71.4%) | 4 (36.4%) |
|  | **C-section** | 6 (60%) | 2 (28.6%) | 7 (63.6%) |
| **PROM** | **Yes** | 3 (30%) | 4 (57.1%) | 6 (54.5%) |
|  | **No** | 7 (70%) | 3 (42.9%) | 5 (45.5%) |
|  |  | Mean ± SD | Mean ± SD | Mean ± SD |
| **Birth gestational age (week)** | | 31.6 ± 1.9 | 31.3 ±1.9 | 31.1 ±1.7 |
| **Birth weight (g)** | | 1549.5 ± 518.3 | 1464.7 ± 495.7 | 1424.1 ± 390.5 |
| **Antibiotic use (day)** | | 1.9 ± 1.4 | 2.3 ± 1.5 | 2.8 ± 3.95 |
| **Gini-Simpson** | | 0.36 ± 0.23 | 0.59 ± 0.19 | 0.65 ± 0.18 |

**S1 Table.**

Infant Demographic and Clinical Characteristics among the Three Types of Gut Microbial Composition

Note: PROM = premature rupture of membranes; MBM = mother’s own breastmilk feeding; Non-MBM = non-mother’s own breastmilk feeding including human donor’s milk and formula feeding
