# Supplementary material for: Gut Microbiome Developmental Patterns in Early Life of Preterm Infants: Impacts of Feeding and Gender
Source: PLoS One. 2016 Apr 25;11(4):e0152751. doi: 10.1371/journal.pone.0152751 (PMC4844123; doi:10.1371/journal.pone.0152751)
Supplement: Supplementary file 3 [file pone.0152751.s004.docx]

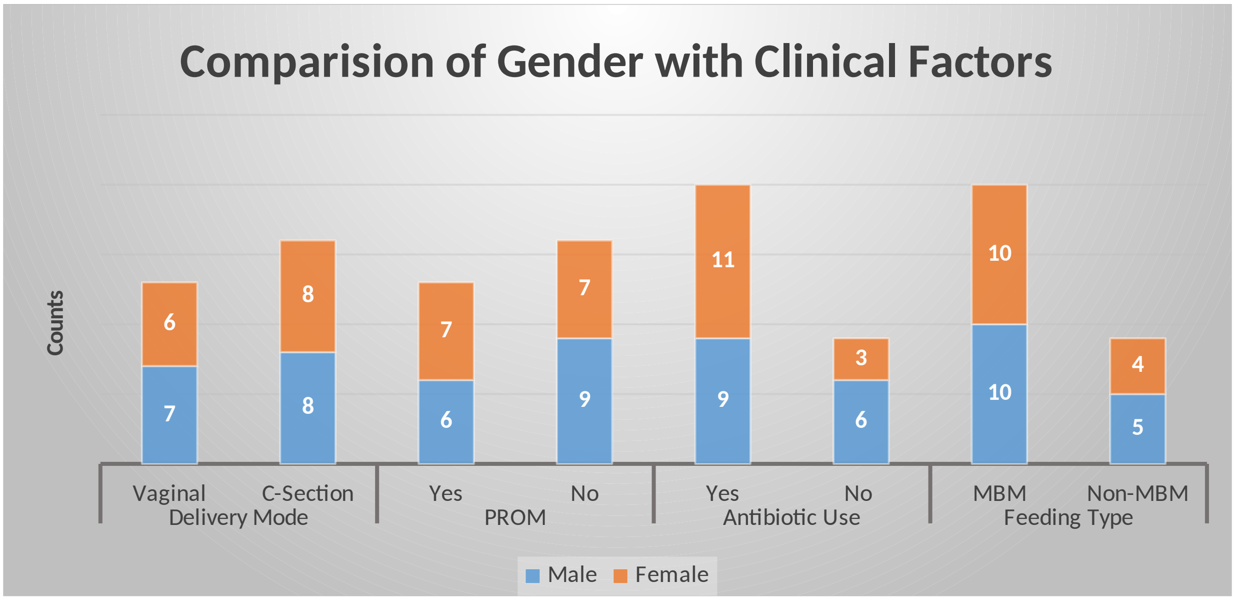

Comparision of Gender with Clinical Factors
Counts
6
7
8
8
7
6
7
9
11
9
3
6
10
10
4
5
Vaginal
C-Section
Delivery Mode
Yes
No
PROM
Yes
No
Antibiotic Use
MBM
Non-MBM
Feeding Type
Male
Female
