# Supplementary material for: Gut Microbiome Developmental Patterns in Early Life of Preterm Infants: Impacts of Feeding and Gender
Source: PLoS One. 2016 Apr 25;11(4):e0152751. doi: 10.1371/journal.pone.0152751 (PMC4844123; doi:10.1371/journal.pone.0152751)
Supplement: Supplementary file 4 [file pone.0152751.s005.docx]

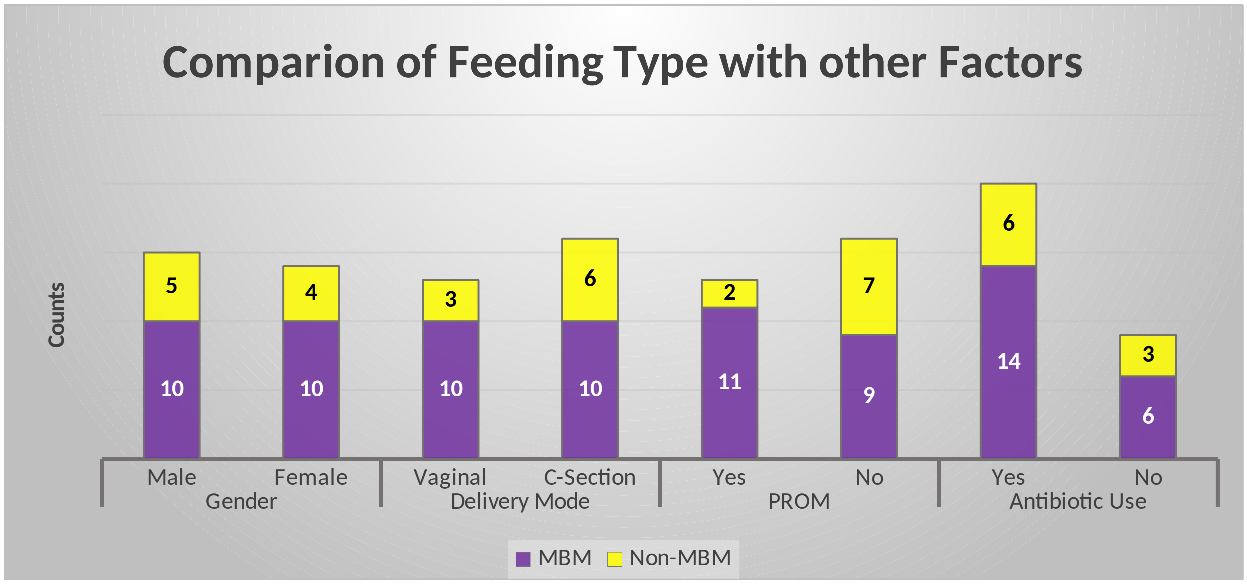

Comparion of Feeding Type with other Factors
Counts
5
4
3
6
2
7
6
3
10
10
10
10
11
9
14
6
Male
Female
Gender
Vaginal
C-Section
Delivery Mode
Yes
No
PROM
Yes
No
Antibiotic Use
MBM
Non-MBM
